# Investigation into the Trampoline Dynamic Characteristics and Analysis of Double Bounce Vibrations

**DOI:** 10.3390/s22082916

**Published:** 2022-04-11

**Authors:** David Eager, Shilei Zhou, Karlos Ishac, Imam Hossain, Adam Richards, Lisa N. Sharwood

**Affiliations:** 1 Faculty of Engineering and Information Technology, University of Technology Sydney, Sydney 2007, Australia; david.eager@uts.edu.au (D.E.); karlos.ishac@uts.edu.au (K.I.); mdimam.hossain@uts.edu.au (I.H.); lisa.sharwood@sydney.edu.au (L.N.S.); 2 Mr Trampoline, 966 Dandenong Road, Melbourne 3163, Australia; adam@mrtrampoline.com.au; 3 Faculty of Medicine and Health, University of Sydney, Sydney 2006, Australia; 4 Faculty of Medicine and Health, University of New South Wales, Sydney 2032, Australia

**Keywords:** trampoline safety, double bounce, kinetic and potential energy transfer, injury prevention, risk management, adventure sport, adolescent play, children’s play

## Abstract

Double bounce is an unusual and potentially very hazardous phenomenon that most trampoline users may have experienced, yet few would have really understood how and why it occurs. This paper provides an in-depth investigation into the double bounce. Firstly, the static and dynamic characteristics of a recreational trampoline are analysed theoretically and verified through experiments. Then, based on the developed trampoline dynamic model, double bounce simulation is conducted with two medicine balls released with different time delays. Through simulation, the process of double bounce is presented in detail, which comprehensively reveals how energy is transferred between users during double bounce. Furthermore, the effect of release time delay on double bounce is also presented. Finally, we conducted an experiment which produced similar results to the simulation and validated the reliability of the trampoline dynamic model and double bounce theoretical analysis.

## 1. Introduction

The trampoline is an item of both recreational and professional sports equipment that is enjoyed by people from different ages, areas, and backgrounds [1,2,3,4]. It also serves as an effective exercise tool to improve health and fitness, with significant energy expenditure during jumping [5]. The benefit of trampolining in improving balance control has been previously reported [6,7]. Trampolines are also used for the training intervention of motor and balance ability of children with intellectual disability (ID); randomised controlled experiments demonstrate significantly improved motor performance, as well as enjoyment, with trampoline training among children with ID [8]. Conversely, there are risks when engaging in physical activity, especially when that activity effectively amplifies the physical abilities of its users [9]. Excessive acceleration and jerk [10] can be causal mechanisms for injurious outcomes of these activities, where sudden impact forces might occur, such as in roller coasters [11], martial arts [12], motorcycling [13], acrobatic gymnastics [14], and children’s playgrounds [15,16,17].

While most injuries in trampolining are caused by dismounting, impacting the trampoline frame, colliding with nearby objects, and becoming caught in the springs [18,19,20,21,22,23,24], a lesser reported risk relates to the excessive impact forces caused by gravitational acceleration of the user as they fall from height above the trampoline. For single users, the risk of serious impact can be mitigated if the users can control their bounce height and landing posture [25]. However, injuries associated with shared use of a trampoline are more difficult to prevent because the bounce height and interaction with the trampoline of one user are strongly affected by the kinetic energy associated with other users [26,27]. It is reported that over 30% of the detailed injury cases are the result of multiple user injuries [28]. The Australian Standard for Indoor Trampoline Facilities [29] expressly recommends only one user per trampoline, for the prevention of such injuries. However, on the backyard trampoline, this is impossible to impose.

A common pattern of shared use of trampolines is the double bounce, where the trampoline is shared by two people. In double bounce, one user can transfer his/her kinetic energy to the other user when the force of their impact pushes the trampoline down and the trampoline gains elastic energy. This energy can be transferred to the other user’s kinetic energy when the trampoline moves back up. In addition, the potential energy of one user could be transferred by landing on the trampoline earlier than the other user. Both the transferred kinetic and potential energy augments the other user’s bounce and sends them higher as a result. Observations indicate that under certain conditions, particularly high energy transfer from one user to another results, leading to surprisingly higher rebound heights, and consequently larger acceleration and jerk than a “normal” bounce [30]. This combination of energy, force, and acceleration imposes higher risks of landing and compression injuries [31].

Due to the unpredictable nature of double bounce, users are often unable to anticipate its occurrence, are caught “off guard”, and therefore are physically unprepared for the immense acceleration forces generated by the energy transfer between users [32]. Furthermore, if the trampoline surface is stretched by one user and has a slope, a shearing force could be introduced to the other user when they contact the trampoline and this may increase the physical injuries [33]. If double bounce is correctly performed by professional players, it can bring a lot of enjoyment to the players and audiences, such as is used in high-performance acrobatic circus acts. An example of multiple people sharing a trampoline is shown in the performance from “The Life Of The Artists” from the Cirque du Soleil Show “KURIOS” [34]. They used the double bounce technique to propel a performer high above the arena. However, for the general user (non-professionals or novices), the double bounce is potentially hazardous and risks serious injury.

Many of the commercial trampoline parks provide their own safety guidelines as a condition of entry for patrons to be allowed use of the facilities, where double bounce is often prohibited. The Australian Standard for Indoor Trampoline Facilities equally articulates the risk management guideline of only one user to be permitted per mat. However, there are no such restrictions when it comes to private and home use [24]. Additionally, anecdotal evidence suggests that some trampoline owners believe it is safe to have numerous children on a single trampoline at one time. In fact, in many cases it will be a parent jumping with their child, which increases the weight difference between users, resulting in a much more unequal weight differential and augments the exchange of energy [35].

Despite this, double bounce is frequently performed and injuries caused by it are continuously reported [36]. A scientific understanding of the double bounce phenomenon’s characteristics has not previously been researched. It therefore remains unclear exactly how the energy is transferred between users during double bounce, or what the main factors are which affect the energy transfer level and process.

To this end, this paper aims to analyse trampoline double bounce dynamics and explore the combination of conditions which would allow the higher injury risk, high energy transfer rates between users. The main contributions of this research include the following. Firstly, the trampoline dynamic model (consisting of stiffness, damping, air resistance, and friction) is built and verified by experiments. With this model, the trampoline force can be accurately described as to when it has deflection and speed. Then, the double bounce is simulated based on the model, and the energy transfer process in double bounce is demonstrated in detail. The gap in the current evidence that is addressed by this study is the demonstration of how the energy is transferred among potential energy, elastic potential energy, and kinetic energy during a double bounce. These findings therefore aid in understanding the double bounce phenomena, but also help to understand other energy transfer scenarios on a trampoline.

## 2. Trampoline Static and Dynamic Characteristics Analysis

**Figure 1:**
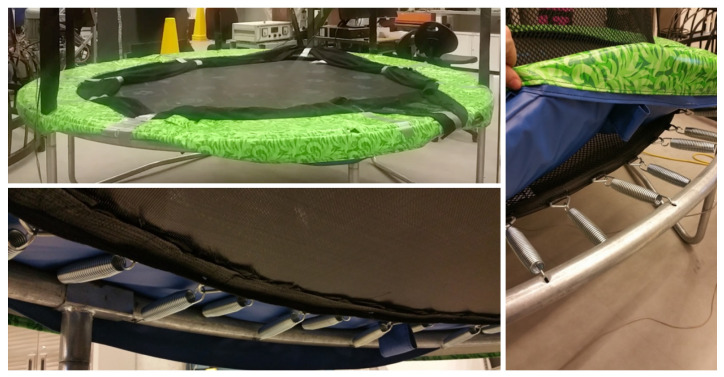
Details of the trampoline used in this research.

In this research, a round trampoline is used for the double bounce analysis, as shown in Figure 1. For this trampoline, the mat is hooked and connected to the frame by springs which are evenly distributed around the frame and have initial extensions which stretch the mat. Springs hold the trampoline mat in place and absorb the displacement of the mat when a jumper impacts and pushes down the mat. The spring recoil then releases that force back into the upward movement of the mat, which sends the jumper back up with a large portion of their landing energy. The main trampoline dimensions and parameters are shown in Table 1.

The springs used on the trampoline are identical double stage springs and the stiffness of a single spring is obtained through testing:

(1) $$k_{s}=\left\{\begin{array}{cc} 96,000\;N/m, & \Delta L<1.5\;\mathrm{mm}\\ 2700\;N/m, & \Delta L\geq 1.5\;\mathrm{mm} \end{array}\right.$$

where $k_{s}$ is spring stiffness and ΔL is spring extension.

### 2.1. Trampoline Static Characteristics

**Figure 2:**
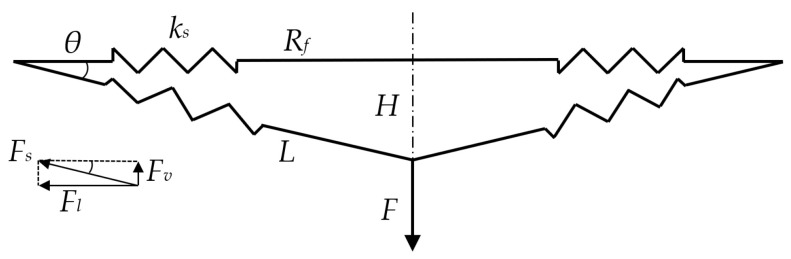
Sketch model of the trampoline under external force.

A simplified model is used to illustrate the trampoline deflection under an external force *F*, as shown in Figure 2. Since the mat is significantly less elastic than the spring, the external force mainly causes spring extension. When the external force acts at the centre of the mat, the springs are stretched and the mat centre moves downward with a height of *H*. At the static state, the external force is balanced by the spring force.

From the initial free state to the new static state under *F*, the spring deformation is:

(2) $$\Delta L=L-R_{f}=\sqrt{R_{f}^{2}+H^{2}}-R_{f}$$

The spring force generated by its deformation is:

(3) $$F_{s}=k_{s}\Delta L$$

The spring forces can be split into vertical force and lateral force. As all the springs are identical and evenly arranged around the circular trampoline frame, the lateral forces are considered to be balanced with each other. As such, only the vertical force acts on the mat. The single spring vertical force $F_{v}$ is:

(4) $$F_{v}=F_{s}sin\theta$$

At the static state, the combined vertical force of all the springs should be equal to the external force but with the opposite direction, and the following equation is obtained:

(5) $$F_{t}=-F=\sum_{i=1}^{N_{s}}F_{vi}=N_{s}k_{s}\left(\sqrt{R_{f}^{2}+H^{2}}-R_{f}\right)\frac{H}{\sqrt{R_{f}^{2}+H^{2}}}$$

where $F_{t}$ is the combined vertical force of all the springs.

**Figure 3:**
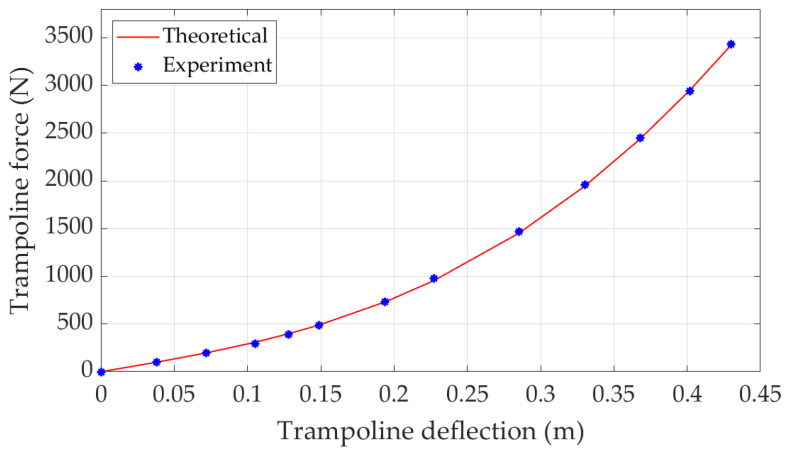
Trampoline static characteristics.

Equation (5) describes the relationship of the trampoline vertical force and its deflection. In real practice, this equation describes the trampoline supporting force to the player when the trampoline is pushed downward with a height of *H*. To verify the static trampoline model, static experiments were conducted. Different masses were applied on the trampoline and its deflections were measured. The experiment results are shown in Table 2. The theoretical analysis and experimental results are compared in Figure 3. It can be seen that the theoretical and experimental results closely match, which confirms that the static trampoline model correctly describes the trampoline static characteristics.

### 2.2. Trampoline Dynamic Characteristics

During the bounce process, the trampoline force is caused by both deflection and velocity. The force caused by deflection is described by Equation (5). For the velocity-induced force, firstly, there is air resistance acting on the trampoline mat surface, which is proportional to squared velocity. Assuming the trampoline centre velocity is *v*, the mat is divided into multiple rings, where the velocity of each ring is:

(6) $$v_{r}=\frac{R_{f}-x}{R_{f}}v$$

where *x* is the distance from the ring to the trampoline centre.

Then, for each ring, the air resistance in the vertical direction is:

(7) $$F_{ar}=\frac{R_{f}}{\sqrt{R_{f}^{2}+H^{2}}}C_{a}{\left(\frac{R_{f}-x}{R_{f}}v\right)}^{2}2\pi xdx$$

where $C_{a}$ is the air resistance coefficient.

The total air resistance of the whole mat would be the integration of the air resistance of all rings:

(8) $$\begin{array}{cc} F_{a} & =\int_{0}^{R_{m}}\frac{R_{f}}{\sqrt{R_{f}^{2}+H^{2}}}C_{a}{\left(\frac{R_{f}-x}{R_{f}}v\right)}^{2}2\pi xdx=\frac{2\pi C_{a}}{R_{f}\sqrt{R_{f}^{2}+H^{2}}}\left(\frac{R_{m}^{4}}{4}-\frac{2R_{f}R_{m}^{3}}{3}+\frac{R_{f}^{2}R_{m}^{2}}{2}\right)v^{2} \end{array}$$

There is also damping resistance working on the trampoline which could be described by an equivalent damping coefficient multiplying the trampoline centre velocity:

(9) $$F_{d}=C_{d}v$$

where $C_{d}$ is the equivalent damping coefficient.

There is also resistance caused by friction between the mat, springs, and frame, which is insignificant compared to the air and damping resistances. It can be described by a constant resistance $F_{c}$.

With the above analysis of the trampoline spring force and resistance, when a player comes into contact with, and moves on, the trampoline, the dynamics of the player can be described by:

(10) $$\left(M_{b}+M_{m}\right)a=F_{t}-M_{b}g-\left(F_{a}+F_{d}+F_{c}\right)sgn\left(v\right)$$

where $M_{b}$ is the player mass, *a* is player acceleration, and *g* is the gravity acceleration, which is 9.81 m/s${}^{2}$ in this research.

When the player leaves the trampoline and moves in the air, it bears air resistance and gravity force. However, the air resistance is much smaller than the gravity force with low velocity and small windward area; thus, it is ignored for the convenience of modelling. The player’s acceleration in the air is:

(11) a=−g

To verify the trampoline dynamic characteristics analysis, the simulation and experiments are conducted with a 15 kg ball bouncing on the trampoline. The simulation model is built in Matlab/Simulink with the trampoline dynamic parameters in Table 3.

**Figure 4:**
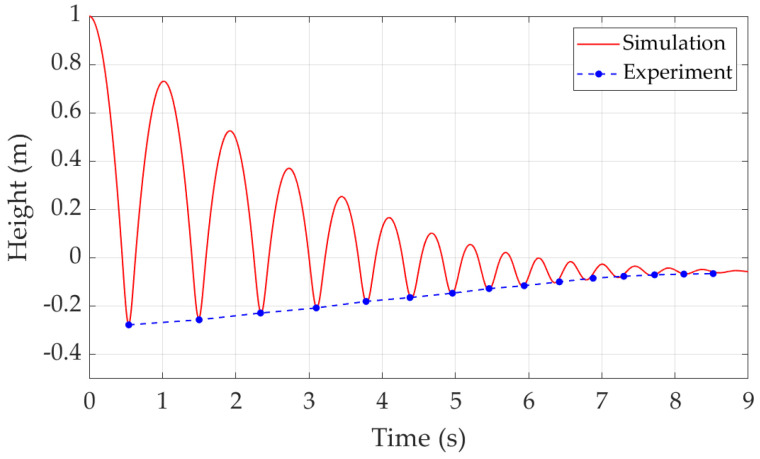
Trampoline dynamic characteristics.

In this case, the ball is released from 1 m above the trampoline and then experiences multiple bounces with decayed heights until it stops on the trampoline. The results are shown in Figure 4. In Figure 4, when the ball height is greater than 0 m, it means the ball is above the mat and moves in the air. When the ball height is less than or equal to 0 m, the ball makes contact with the mat and moves with the mat together. The final negative ball height means the ball stays on the mat statically and the mat is pushed downward with the extended springs providing vertical force to support the the ball mass.

**Figure 5:**
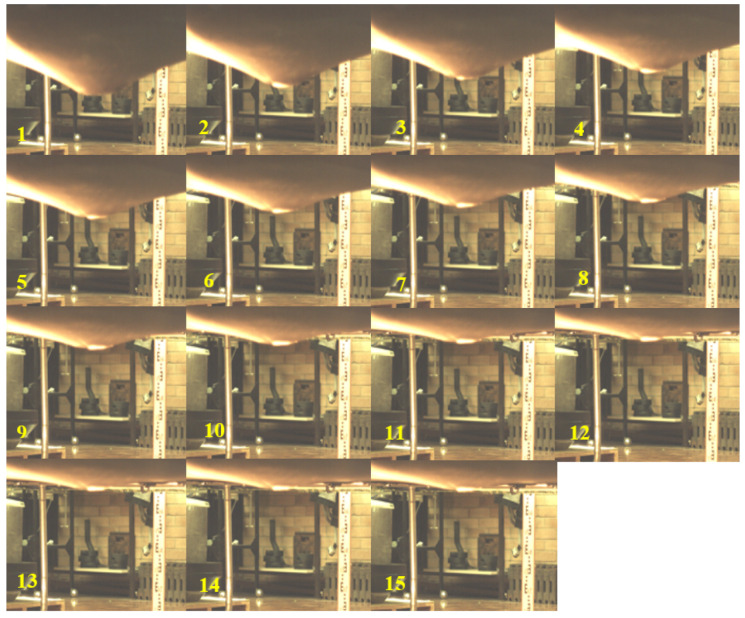
High-speed camera footage of trampoline dynamic characteristic experiment.

In the experiment, the exact displacement and timestamp of the ball on the trampoline was recorded with the use of a high-speed camera. The displacements and moments when the ball reaches the bottom points are identified from the video, as shown in Figure 5. At these points, the trampoline experiences its maximum deflections. Between every two bottom points, the ball completes a bounce. The trampoline deflection and time duration of each bounce is listed in Table 4.

The experiment results are also presented in Figure 4 with blue dots connected by the blue dashed line. The blue dots represent the values in Table 4. The blue dashed line shows the overall trend of the lowest points of every bounce. Comparing the simulation and experiment results, the trampoline dynamic model correctly describes the dynamics of a ball bouncing on the trampoline.

## 3. Double Bounce Theoretical Analysis

In this section, double bounce of two balls are analysed based on the developed trampoline dynamic model as an example to demonstrate how energy is transferred during double bounce. The two balls used for analysis are 20 kg (Ball 1) and 15 kg (Ball 2), respectively. Based on the verified trampoline dynamic model in Section 2, two balls are released from the same height, 1 m above the trampoline, but at slightly different times. Ball 2 is released after Ball 1 with variable time delays which bring different energy transfer scenarios.

**Figure 6:**
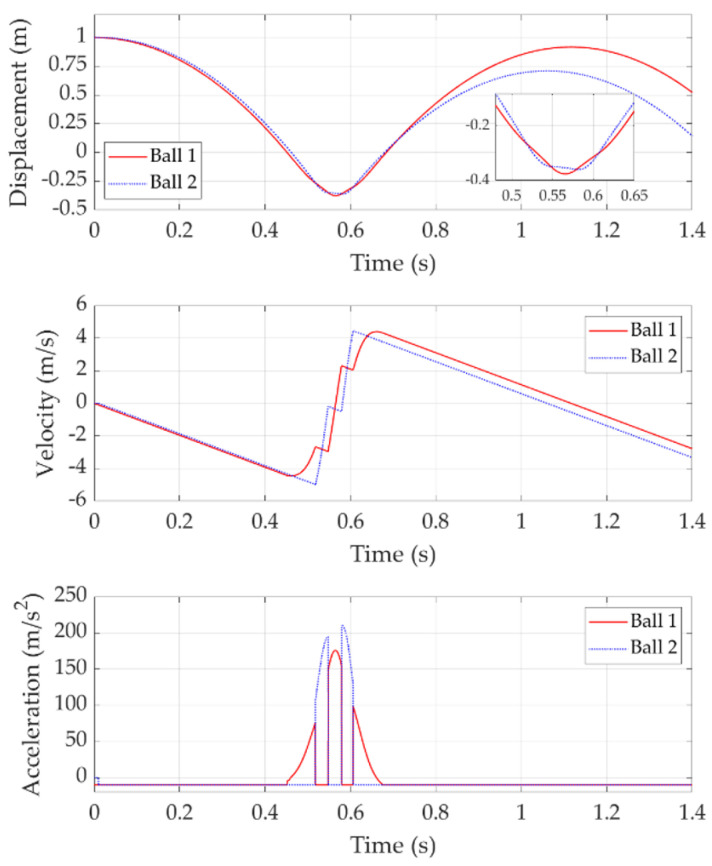
Double bounce with 0.01 s release time delay.

Figure 6 shows the double bounce with a release time delay of 0.01 s. In this case, Ball 2 is released 0.01 s after Ball 1. After released, Ball 1 experiences free fall until it hits the trampoline at about 0.45 s. At this stage, the trampoline moves downwards with Ball 1 together and its deflection generates resistance to decelerate Ball 1. When the trampoline resistance is greater than Ball 1 gravity, Ball 1 acceleration changes to positive values.

Ball 2 is released just 0.01 s after Ball 1 and follows Ball 1 with free fall. When Ball 1 is decelerating but lower than Ball 2, Ball 2 does not contact with the trampoline and still has free fall. However, when Ball 2 is lower than Ball 1 and contacts with the trampoline, it replaces Ball 1 to push the trampoline downward and its deceleration is affected by the trampoline deflection and velocity. At this time, Ball 1 is above Ball 2 with negative velocity, which means free fall happens again to Ball 1. Decelerating Ball 2 and free fall Ball 1 will cause Ball 1 to be lower than Ball 2 again. Then, Ball 1 and Ball 2 change their roles to push the trampoline downward. They repeat the process until the trampoline reaches its bottom point when it has the maximum deflection.

When Ball 1 makes contact with the trampoline at the bottom point, its velocity is 0 m/s. Ball 2 moves down with a very small velocity at this time. Then, Ball 1 starts to bounce up with the positive velocity. During the ascending process, the trampoline meets Ball 2 and is obstructed by Ball 2. Ball 2 obtains the trampoline force which provides it with the positive acceleration. However, Ball 1 loses the trampoline force and decelerates under the effect of gravity. During the Ball 2 ascending process, the trampoline is also obstructed by Ball 1. They repeat the process until both of them leave the trampoline and the trampoline returns to its free state.

With the multiple rotations of actions between the two balls, it is hard to predict which ball will obtain more energy and bounce higher. This result indicates that in real practice, if two players land on the trampoline with a very short time delay, energy transfer can happen in both directions, thus either of them can bounce higher than expected.

**Figure 7:**
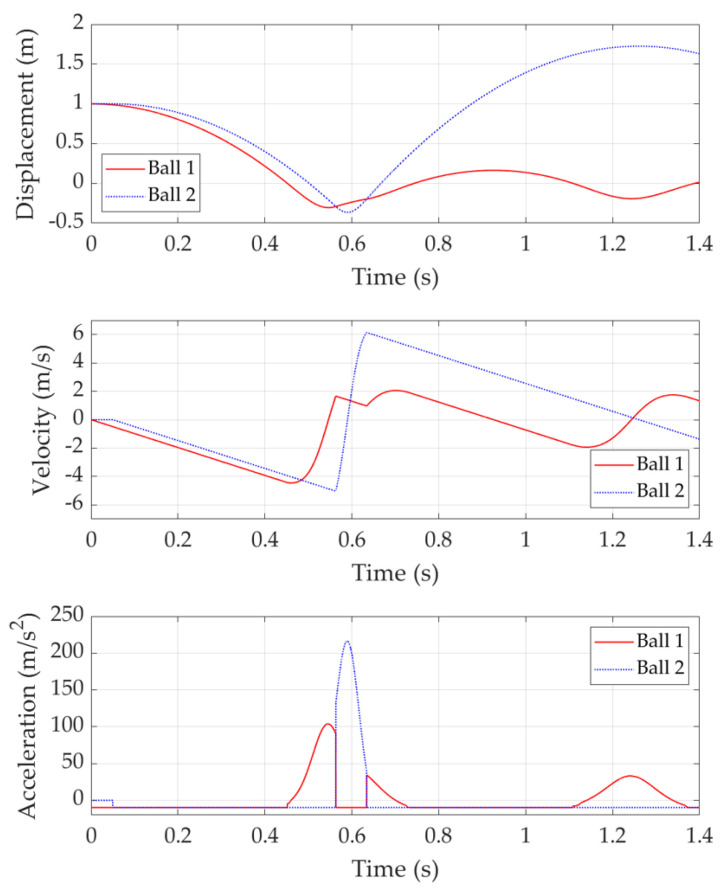
Double bounce with 0.05 s release time delay.

Figure 7 shows the double bounce with a release time delay of 0.05 s. In this case, Ball 2 is released 0.05 s after Ball 1. Similarly, Ball 1 hits the trampoline first and bears the trampoline resistance. Before Ball 2 comes, Ball 1 reaches the bottom point and its velocity is 0 m/s at this point. All the Ball 1 potential energy is transferred into the trampoline spring elastic potential energy except for those lost due to resistance. Then, Ball 1 moves upward under the spring force. However, Ball 1 only obtains the chance to move up for a very short time period, then Ball 2 comes and pushes the trampoline down to another bottom point. It is worth noting that this bottom point is lower than the previous result and most of the previous spring elastic potential energy is kept and will be transferred to Ball 2 kinetic energy later.

During the Ball 2 ascending process, the trampoline makes contact with Ball 1 and starts to accelerate Ball 1, while Ball 2 starts to decelerate due to gravity. However, most of the spring elastic potential energy has been transferred to Ball 2, which brings Ball 2 a high velocity of around 6 m/s. As a comparison, Ball 1 only obtains a velocity of around 2 m/s when it finally leaves the trampoline. This means the trampoline’s elastic energy is mostly transferred into Ball 2 kinetic energy; thus, Ball 2 obtains a much higher bounce than Ball 1. In other words, energy is transferred from Ball 1 to Ball 2 through the trampoline.

**Figure 8:**
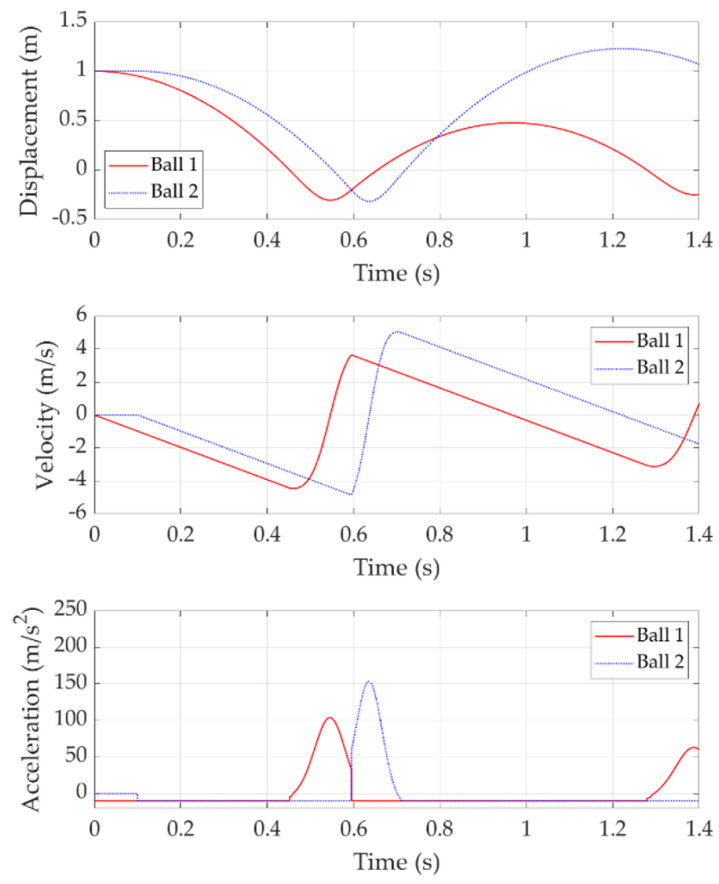
Double bounce with 0.1 s release time delay.

Figure 8 shows the double bounce with a release time delay of 0.1 s. In this case, Ball 2 is released 0.1 s after Ball 1. Ball 1 firstly pushes the trampoline to its bottom point and bounces upwards. When Ball 2 makes contact with the trampoline during the Ball 1 ascending stage at around 0.6 s, most of the trampoline elastic energy has been transferred to Ball 1 kinetic energy, as demonstrated by the around 3.5 m/s velocity of Ball 1. Therefore, Ball 2 only takes advantage of part of the trampoline-stored elastic potential energy from Ball 1, which appears as a limited energy transfer between two balls.

**Figure 9:**
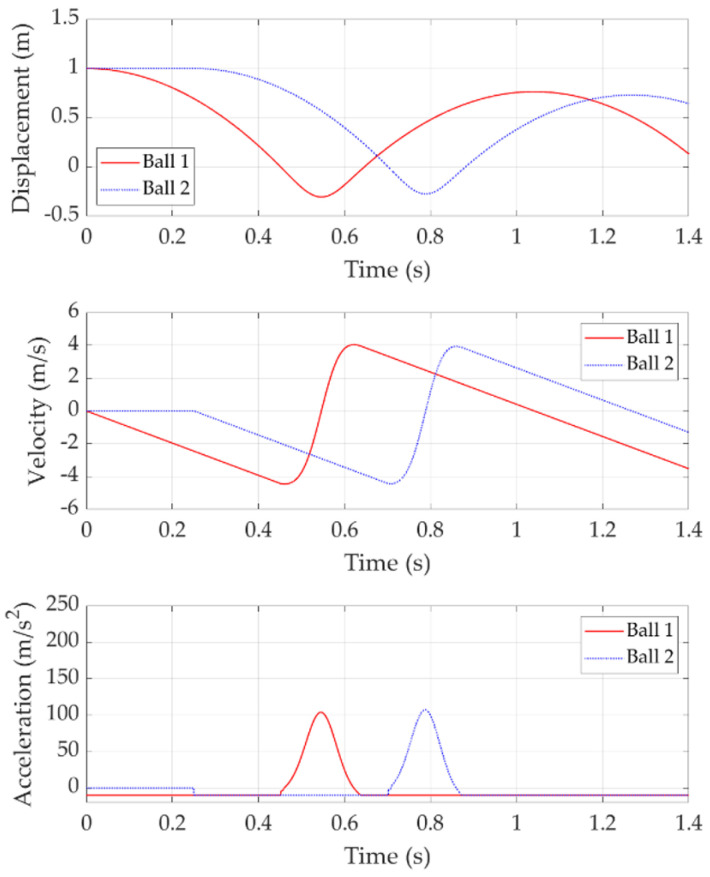
Double bounce with 0.25 s release time delay.

Figure 9 shows the double bounce with a release time delay of 0.25 s. In this case, Ball 2 is released 0.25 s after Ball 1. Ball 1 finishes its free fall and bounce before Ball 2 reaches trampoline. Thus, no energy is transferred between the two balls. This case is equivalent to single bounce of two balls.

**Figure 10:**
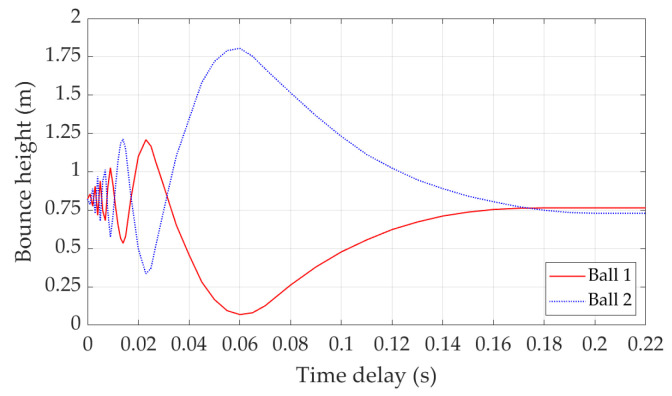
Bounce height of balls with different release time delay.

The above analysis demonstrates that the energy transfer during double bounce is related to the release time delay of two balls. Figure 10 summarises the bounce heights of two balls with different release time delays. The results show that when the release time delay is less than 0.031 s, energy transfer could happen in both directions even if Ball 1 is released first. Figure 6 is representative of these scenarios. When the release time delay is greater than 0.031 s, energy is transferred from Ball 1 to Ball 2. The amount of transferred energy is determined by the release time delay. Figure 7 is representative of the scenarios with release time delay between 0.031 s and 0.06 s. Figure 8 is representative of the scenarios with release time delay larger than 0.06 s. When the release time delay is greater than 0.2 s, two balls make contact with the trampoline at different times; thus, no energy is transferred between them. Figure 9 is representative of these scenarios.

Comparing the accelerations of different scenarios, it could be found that double bounce significantly increases the balls’ accelerations at the bottom points of the trampoline, which are much larger than the measured accelerations in a normal single player bounce [37,38]. This result indicates that the increased acceleration and impact force during double bounce can go beyond the trampoline manufacturer provided force and acceleration range and could cause serious injuries to human users [39].

## 4. Double Bounce Experiment

### 4.1. Experiment Setup and Methodology

**Figure 11:**
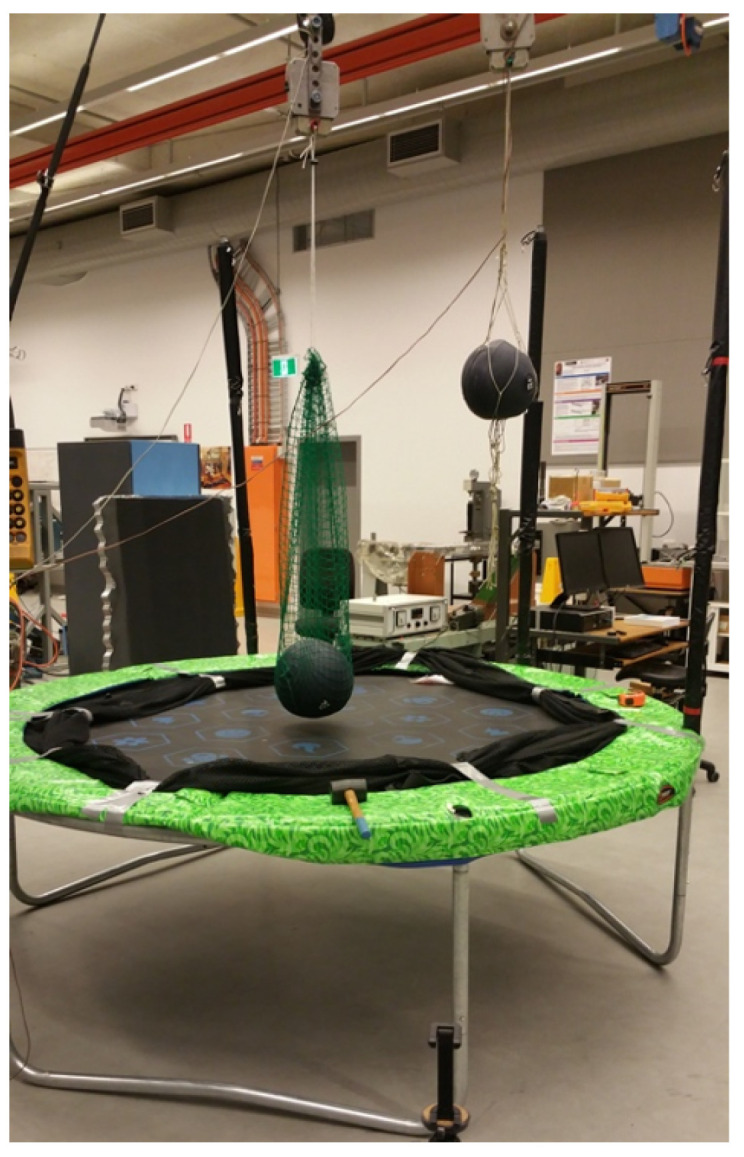
Experiment setup depicting the two medicine balls and their respective bomb release mechanisms.

In this experiment, two medicine balls were used and dropped above the trampoline with varying release time delays to verify the theoretical analysis results. The setup equipment for this experiment is shown in Figure 11. Ball 1 is 20 kg and Ball 2 is 15 kg, the same values as in the simulation model.

Without a reliable means of electronically controlling the delay between releases, a variety of near-similar experiments are conducted through the control of human timing, i.e., the releases are physically triggered by an individual over and over again. Since the data are being collected through video recordings, a slowed-down observation of the footage provided a fairly accurate reading of the time delays, and with enough repeated tests, a variety of different time delays are observed, as well as the corresponding test results. The video recording is made with 60 frames per second recording, which provides a time resolution of 0.0167 s.

To successfully carry out this aspect of the experiment, the following sequence process was followed:

1. Ensure the balls are stationary in their initial heights above the trampoline.
2. Begin video recording.
3. Release Ball 1.
4. Release Ball 2 nearly immediately after the Ball 1 release.
5. Wait for balls to settle on trampoline.
6. End video data collection.

**Figure 12:**
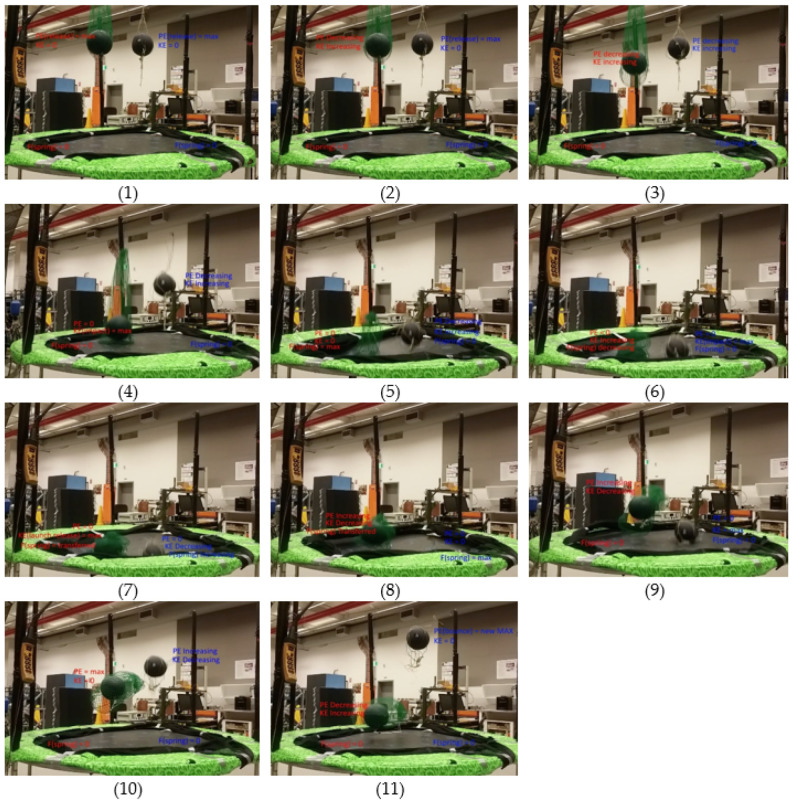
Double bounce process demonstration as recorded during experiment.

The detailed sequence process of one of the double bounce experiments is shown in Figure 12, as an example. It can be seen that Ball 1 is released first and Ball 2 is released after. These pictures clearly demonstrate the energy transfer in double bounce where Ball 1 bounce height is much lower than Ball 2.

### 4.2. Experiment Results Analysis

In this experiment, a single bounce of two balls was firstly examined. Two balls were released individually above the trampoline and their first bounces, which represent the highest bounce, were recorded. The single bounce is also simulated with the proposed trampoline dynamic model. The experiment and simulation results are shown in Table 5. The results show that for both balls, the experiment and simulation bounce height errors are less than 2%, which demonstrates that the simulation model can describe the ball bounce with great accuracy.

The double bounce experiment and simulation results are shown in Table 6. The single bounce simulation results are also presented, which works as a benchmark to assess the energy transfer between two balls during double bounce.

**Figure 13:**
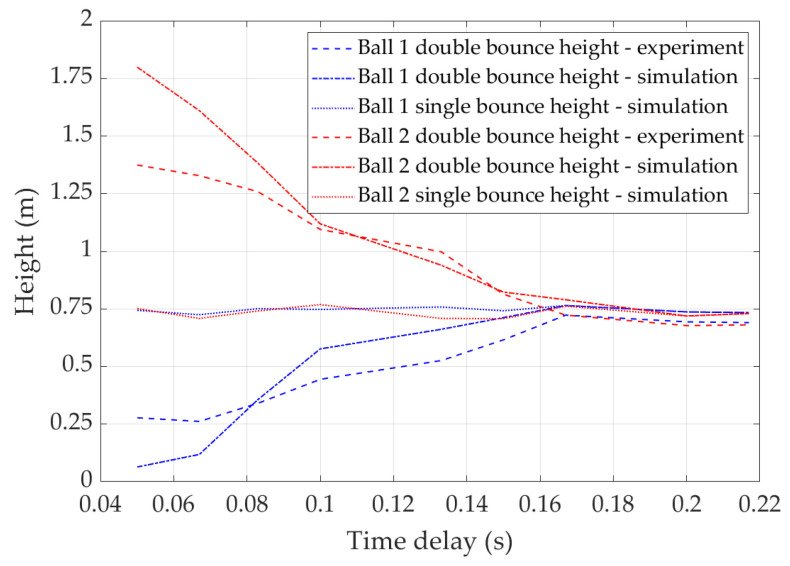
Bounce height of balls with different release time delay.

Figure 13 illustrates the double bounce heights of two balls. In this figure, the *x* axis is the release time delay between the balls for the convenience of demonstrating the relationship between energy transfer and release time delay. As a comparison, the single bounce height is also presented. Due to the experiment condition limitations, the achieved minimum time delay is 0.05 s. It can be observed that the experiment results and simulation results share similar trends.

**Figure 14:**
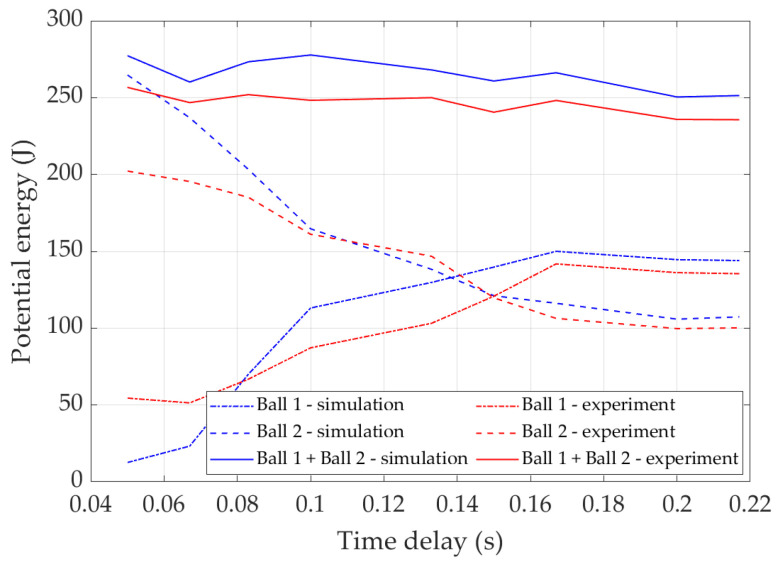
Potential energy of balls after double bounce.

Taking both the mass and height into account, the potential energy of two balls after double bounce occurs is calculated and shown in Figure 14. This figure correctly shows the trend of potential energy regarding release time delay.

**Figure 15:**
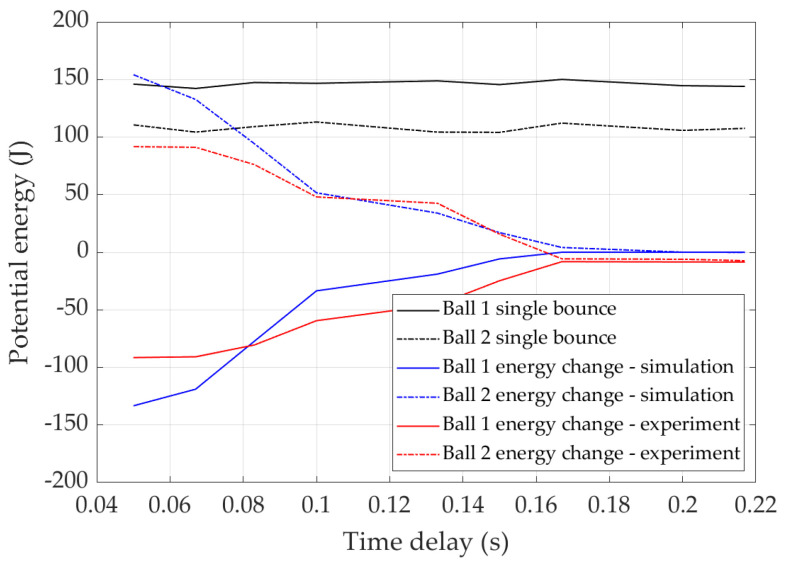
Potential energy comparison of double bounce and single bounce.

Figure 15 shows the potential energy change during double bounce and its comparison with single bounce, which is calculated by the difference between the double bounce potential energy and the single bounce potential energy. In the single bounce, the potential energy after bounce is only reduced by the air resistance, damping, and friction. It is shown that the potential energy change decreases with the increase of release time delay.

## 5. Conclusions

The double bounce manoeuvre creates energy transfer from one trampoline user to another and is frequently employed in everyday trampolining as well as high-performance acrobatics. The energy forces occurring in this move impose high risk to the users, and this has not been comprehensively researched. In this study, we have analysed the mechanical and kinetic energy properties of the double bounce phenomena based on a typical trampoline. We firstly developed and proposed a trampoline dynamic model by considering the spring characteristics, air resistance, damping, and friction. The trampoline model is verified through our conducted experiments. Then, we examined the characteristics of double bounce through both simulation and experiment. Our results clearly demonstrated how the energy is transferred between two users and how the release time delay affects the double bounce.

The pattern of energy transfer between two trampoline users is determined by many conditions. These can include variables such as trampoline characteristics, users’ weight and fall height, fall time delays, and any additional energy applied by users actively extending their legs to push the trampoline further when they land by changes in their centre of gravity. The interplay of these factors and their respective magnitudes are research questions that should inspire future work on this topic. These findings may also provide reference for energy transfer analyses between users in other conditions.

In this study we have shown that the energy transfer between two objects impacting a trampoline surface is affected by time delay in addition to the trampoline characteristics. These results provide evidence-based encouragement for trampoline manufacturers to test the double bounce time delay across an acceptable range of user weights and heights to determine the energy transfer in potential double bounce phenomenon for their product. It would then be possible to make design changes that would retain the enjoyment factor for users but reduce the energy transfer and in so doing reduce the risk of injury.

## Figures and Tables

**Table 1:** Trampoline dimensions and parameters.

Parameter	Description	Value	Unit
$R_{m}$	Mat radius	1	m
$R_{f}$	Frame radius	1.2	m
$H_{m}$	Mat height	0.735	m
$M_{m}$	Mat mass	1	kg
$N_{s}$	Number of springs	60	-

**Table 2:** Trampoline static deflection under different external force.

Mass (kg)	Equivalent Force (N)	Trampoline Deflection (m)
0	0	0
10	98.1	0.038
20	196.2	0.072
30	294.3	0.105
40	392.4	0.128
50	490.5	0.149
75	735.8	0.194
100	981.0	0.227
150	1471.5	0.285
200	1962.0	0.330
250	2452.5	0.368
300	2943.0	0.402
350	3433.5	0.430

**Table 3:** Trampoline dynamic parameters.

Parameter	Value	Parameter	Value
$C_{a}$	Air resistance coefficient	2.3	Ns${}^{2}$/m${}^{4}$
$C_{d}$	Damping coefficient	16	Ns/m
$F_{c}$	Friction force	2.5	N

**Table 4:** Trampoline dynamic characteristics experiment result.

Number	Deflection (m)	Time (s)	Number	Deflection (m)	Time (s)
1	0.277	0.54	9	0.115	5.94
2	0.256	1.50	10	0.099	6.42
3	0.228	2.34	11	0.084	6.88
4	0.207	3.10	12	0.076	7.30
5	0.180	3.78	13	0.070	7.72
6	0.164	4.38	14	0.067	8.12
7	0.146	4.96	15	0.065	8.52
8	0.127	5.46			

**Table 5:** Comparison of experiment and simulation results of single bounce.

Ball Mass	Release Height	Bounce Height (m)	Bounce Height (m)	Error of Experiment
(kg)	(m)	Experiment	Simulation	and Simulation (%)
15	0.974	0.702	0.711	1.28
20	0.972	0.728	0.742	1.92

**Table 6:** Double bounce experiment and simulation results.

Group	B1 Release	B2 Release	Double Bounce	B1 Double Bounce (m)
Number	Height (m)	Height (m)	Time Delay (s)	Experiment
1	0.974	1.028	0.050	0.278
2	0.950	0.972	0.067	0.262
3	0.984	1.014	0.083	0.340
4	0.979	1.050	0.100	0.444
5	0.993	0.972	0.133	0.526
6	0.972	0.969	0.150	0.616
7	1.000	1.042	0.167	0.723
8	0.965	0.986	0.200	0.694
9	0.962	1.000	0.217	0.690
**B2 Double Bounce**	**B1 Double Bounce**	**B2 Double Bounce**	**B1 Single Bounce**	**B2 Single Bounce**
**(m) Experiment**	**(m) Simulation**	**(m) Simulation**	**(m) Simulation**	**(m) Simulation**
1.374	0.064	1.799	0.744	0.751
1.328	0.119	1.610	0.725	0.709
1.258	0.357	1.382	0.751	0.741
1.095	0.577	1.119	0.747	0.768
0.998	0.661	0.940	0.758	0.709
0.814	0.712	0.823	0.742	0.707
0.723	0.764	0.790	0.765	0.762
0.677	0.737	0.719	0.737	0.719
0.681	0.734	0.73	0.734	0.731

B1 represents Ball 1; B2 represents Ball 2.

## Data Availability

The data presented in this study are available on request from the corresponding author.

## Abbreviations

The following abbreviations are used in this manuscript:

B1	Medicine ball 1
B2	Medicine ball 2

## References

[1] Roffe L., Pearson S., Sharr J., Ardagh M. (2018). The effect of trampoline parks on presentations to the Christchurch Emergency Department. N. Z. Med. J..

[2] Choi E.S., Jang J.H., Woo J.H., Choi J.U., Cho J.S., Yang H.J. (2018). Pediatric Trampoline-Related injuries in a nationwide registry in South Korea, 2011 to 2016. Yonsei Med. J..

[3] Lim F.M.T., James V., Lee K.P., Ganapathy S. (2021). A retrospective review of trampoline-related injuries presenting to a paediatric emergency department in Singapore. Singap. Med. J..

[4] Eager D. (2008). Trampolines—Facts and myths. Kidsafe Play. News.

[5] Alexander K., Clement T., Draper N. (2021). Developing a mathematical model to predict energy expenditure while bouncing on a trampoline. Eur. J. Sport Sci..

[6] de Oliveira M.R., da Silva R.A., Dascal J.B., Teixeira D.C. (2014). Effect of different types of exercise on postural balance in elderly women: A randomized controlled trial. Arch. Gerontol. Geriatr..

[7] Hahn J., Shin S., Lee W. (2015). The effect of modified trampoline training on balance, gait, and falls efficacy of stroke patientss. J. Phys. Ther. Sci..

[8] Giagazoglou P., Kokaridas D., Sidiropoulou M., Patsiaouras A., Karra C., Neofotistou K. (2013). Effects of a trampoline exercise intervention on motor performance and balance ability of children with intellectual disabilities. Res. Dev. Disabil..

[9] Davidson P., Wilson S., Wilson B., Eager D., McIntosh A., Chalmers D. (2013). Analysis of energy flow during playground surface impacts. J. Appl. Biomech..

[10] Hayati H., Eager D., Pendrill A.M., Alberg H. (2020). Jerk within the context of science and engineering—A systematic review. Vibration.

[11] Pendrill A.M., Eager D. (2020). Velocity, acceleration, jerk, snap and vibration: Forces in our bodies during a roller coaster ride. Eur. J. Phys..

[12] Ishac K., Eager D. (2021). Evaluating martial arts punching kinematics using a vision and inertial sensing system. Sensors.

[13] Whyte T., Gibson T., Anderson R., Eager D., Milthorpe B. (2015). Mechanisms of head and neck injuries sustained by helmeted motorcyclists in fatal real world crashe—Analysis of 47 in-depth cases. J. Neurotrauma.

[14] Grapton X., Lion A., Gauchard G.C., Barrault D., Perrin P.P. (2013). Specific injuries induced by the practice of trampoline, tumbling and acrobatic gymnastics. Knee Surg. Sports Traumatol. Arthrosc..

[15] Eager D., Hayati H., Chapman C. Impulse force as an additional safety criterion for improving the injury prevention performance of impact attenuation surfaces in children’s playgrounds. Proceedings of the ASME International Mechanical Engineering Congress and Exposition.

[16] Eager D., Hayati H. (2019). Additional injury prevention criteria for impact attenuation surfacing within children’s playgrounds. ASCE-ASME J. Risk Uncertain. Eng. Syst. Part B Mech. Eng..

[17] Barker R., Sharwood L., Eager D. (2019). Ensuring safety in public playgrounds: Everybody’s business. Med. J. Aust..

[18] Eager D., Scarrott C., Nixon J., Alexander K. (2012). Survey of injury sources for a trampoline with equipment hazards designed out. J. Paediatr. Child Health.

[19] Eager D., Scarrott C., Nixon J., Alexander K. (2013). Injury survey of a non-traditional ‘soft-edge’ trampoline designed to lower equipment hazards. Inj. Control Saf. Promot..

[20] Alexander K., Eager D., Scarrott C., Sushinsky G. (2010). Effectiveness of pads and enclosures as safety interventions on consumer trampolines. Inj. Prev..

[21] Eager D., Nixon J., Chapman C. Australian trampoline Standard—Impact attenuation padding systems. Proceedings of the ABC6 Biomechanics.

[22] Eager D., Chapman C., Davidson P. Trampoline frame impact attenuation: Padded metal-frame vs soft-edge system. Proceedings of the IUTAM Symposium on Impact Biomechanics in Sport.

[23] Ashby K., Pointer S., Eager D., Day L. (2015). Australian trampoline injury patterns and trends. Aust. N. Z. J. Public Health.

[24] Sharwood L., Adams S., Blaszkow T., Eager D. (2018). Increasing injuries as trampoline parks expand within Australia: A call for mandatory standards. Aust. N. Z. J. Public Health.

[25] Hossain I., Zhou S., Ishac K., Lind E., Sharwood L., Eager D. (2021). A Measurement of ‘walking-the-wall’ dynamics: An observational study using accelerometry and sensors to quantify risk associated with vertical wall impact attenuation in trampoline parks. Sensors.

[26] Eager D., Chapman C., Matotek A. Double bounce vibration on trampolines and associated injuries. Proceedings of the 12th ICBEN Congress on Noise as a Public Health Problem.

[27] Ashby K., Eager D., D’Elia A., Day L. (2015). Influence of voluntary standards and design modifications on trampoline injury in Victoria, Australia. Inj. Prev..

[28] Routley V. Trampoline Injuries. https://www.monash.edu/__data/assets/pdf_file/0018/218430/haz13.pdf.

[29] (2015). Trampolines for Domestic Use—Safety Aspects.

[30] Eager D., Chapman C., Bondoc K. Characterisation of trampoline bounce using acceleration. Proceedings of the 7th Australasian Congress on Applied Mechanics.

[31] Pendrill A.M., Eager D. (2014). Free fall and harmonic oscillations: Analyzing trampoline jumps. Phys. Educ..

[32] Mulligan C.S., Adams S., Brown J. (2017). Paediatric injury from indoor trampoline centres. Inj. Prev..

[33] Kim K.H., Kim H.S., Kang M.S., Park S.S. (2019). Varus shearing force is a main injury mechanism of pediatric trampoline-related injury in addition to compressive axial loading. PLoS ONE.

[34] du Soleil C. The Life of the Artists. https://www.youtube.com/watch?v=fzwmSwVVFfU.

[35] Hurson C., Browne K., Callender O., O’donnell T., O’Neill A., Moore D.P., Fogarty E.E., Dowling F.E. (2007). Pediatric trampoline injuries. J. Pediatr. Orthop..

[36] Newsroom Maya’s Story: Bouncing Back from a Serious Injury. https://www.acc.co.nz/newsroom/stories/mayas-story/.

[37] Eager D. Accelerometers used in the measurement of jerk, snap and crackle. Proceedings of the Australian Acoustical Society 2018 Annual Conference.

[38] Eager D., Pendrill A., Reistad N. (2016). Beyond velocity and acceleration: Jerk, snap and higher derivatives. Eur. J. Phys..

[39] Brown O.H., Mullineaux D.R., Mulloy F. (2021). Dynamic testing to determine and predict trampoline function. Sports Eng..

